# Judgment of duration and passage of time in prospective and retrospective conditions and its predictors for short and long durations

**DOI:** 10.1038/s41598-022-25913-9

**Published:** 2022-12-23

**Authors:** Natalia Martinelli, Sylvie Droit-Volet

**Affiliations:** Université Clermont Auvergne, LAPSCO, CNRS, F-63000 Clermont-Ferrand, France

**Keywords:** Psychology, Human behaviour

## Abstract

The study investigated participants' verbal duration judgment and judgment of passage of time (PoT) when presented with an image for a few seconds (20 to 45 s) or minutes (80 to 180 s) with prospective and retrospective temporal judgment instruction, with their level of attention devoted to time tested. Their self-reported levels of emotion and attention were also assessed, as well as their individual impulsivity traits. Structural equation analyses showed that the best predictor of PoT judgment was emotion (boredom) regardless of duration range. For duration judgment, the best predictor for short durations was attention-related factors. However, for long durations, these factors ceased to be significant and were replaced by emotion, in the same way as for the PoT judgment. Indeed, these analyses suggested that duration judgment and PoT judgment were related for long durations of more than one minute, whereas they were not related for short durations of a few seconds.

## Introduction

In the field of psychology, a very large number of studies have been published on the judgment of short durations of a few milliseconds or seconds. They have highlighted the main characteristics of the judgment of short durations: scalar properties of time, i.e., time judgments that are on average accurate^1^. They also showed that time distortions can nevertheless occur when attention is diverted from time processing as in the dual-task paradigm, or when the level of arousal is high as in threatening situations. A shortening of time in the attention condition and a lengthening of time in the emotional condition are then observed. An internal clock mechanism was suggested to explain the judgment of short durations and models were proposed. These models were examined and developed by neuroscientists who attempted to understand how the brain processes short durations^2–4^. As the behavioral sciences developed further, psychologists then began to take an interest in and investigate the link between the judgment of durations and the phenomenological time experienced by individuals in daily life. Phenomenological time is expressed in the form of an acceleration or a slowing down of the speed of the passage of time. This judgment of the passage of time (PoT) is generally assessed with a single question (e.g., How do you experience time passing, from very slow to very fast?) and a 7-point Likert-type response scale^5–7^.

Studies on the relationships between duration judgments and PoT judgments are very rare. The few studies that have focused on temporal judgments for short durations in the range of milliseconds and seconds have not found any significant relationship between these two types of temporal judgment, i.e., 0.35–1.650 s^8^, 2–8 and 3–33 s^9^. In line with these findings, the predictors of variance in temporal judgment have been shown to differ between the two types of judgment, being the level of attention directed towards the task for the duration judgment, and the emotion experienced (happiness, sadness, calmness, nervousness) for the PoT judgment. Specifically, variations in the PoT judgment would not result from unconscious primary emotions (e.g., fear) that are known to automatically affect the judgment of short durations by speeding up the internal clock, but from self-conscious emotions (feeling happy, feeling calm). This is consistent with the results of recent Covid studies showing that the conscious emotional state (i.e., happiness, boredom, and life satisfaction) was the main predictor of the slowing down of the passage of time during the lockdown^10–12^.

However, a significant correlation between duration judgments and PoT judgments was found when participants were asked to estimate long durations of several minutes: i.e., 2–8 min^9^, 2–3 and 8–32 min^13^. In addition, for this range of durations, variance in both types of judgments was explained by the same emotional factor, comprising both the level of happiness and that of emotional arousal. Based on these results, Droit-Volet et al.^13^ suggested that PoT judgments and duration judgments of several minutes make use of common memory processes that permit the recovery of information in long-term memory. These processes would be close to those described in the retrospective temporal judgment paradigm in which participants become aware that they should judge the duration of the stimulus only after it has been presented. In this condition, when attention is not directed to time processing, duration is not encoded and time estimates are based on non-temporal information stored and retrieved in memory (e.g., the emotion state experienced during the interval)^14,15^.

Judgments of long durations (min) and PoT judgments are therefore assumed to involve retrospective memory processes, which differ from those involved in the prospective condition (attention, working memory, internal clock) used for the judgment of short durations. When using the prospective paradigm, attention is directed to time processing, and duration is measured by an internal clock system. PoT is indeed thought to be judged retrospectively, that is, once the event has ended. However, the judgment of long durations in the above studies was tested prospectively, with the participants being explicitly instructed to judge the duration of the incoming stimulus. The conditions of temporal judgment (retrospective vs. prospective) therefore differ between these two types of judgment. However, a series of successive temporal trials have been used to assess PoT judgments, thus casting doubt on the reality of a purely retrospective judgment. In a recent study, Martinelli and Droit-Volet^16^ showed that, in a prospective experimental condition, the PoT judgment also varied with temporal information, i.e., when duration was a salient item of information for the task. The PoT was indeed judged to be slower, the longer the stimulus duration was. In fact, the PoT judgment was based on the contextual information that was most salient for the individual (duration, emotion, or task difficulty). This finding is consistent with the contextual self-duration theory of present PoT judgment (see discussion)^7,16^.

The originality of the present study was to further examine in the same experiment the differences between duration judgment and PoT judgment for both short (s) and long (min) durations and in two temporal instruction conditions, i.e., prospective and retrospective, when the participants were or were not asked to be attentive to time. To ensure a truly retrospective judgment, a single time trial was used and their level attention devoted to time was also assessed. On this single trial, participants gave their verbal estimates of the stimulus duration (neutral image) and their PoT judgment. They also answered a series of questions on their subjective experience of non-temporal context, designed to identify the best predictor of each type of judgment for the two duration ranges. The tested predictors of PoT judgment vary across studies suggesting that this temporal judgment is highly context-dependent^7,16^. However, the best predictors of feeling of time passing when viewing a single image might be related to the level of attention directed to the image^8,16^ and emotion (boredom, happiness, sadness, arousal)^10,12,16,17^. In addition, by manipulating the instruction on the duration of the task, Tanaka and Yotsumoto^18^ showed that the PoT judgment for a given task (2-back task) was affected by temporal expectations of events. Accordingly, we also assessed participants’ projection into the future, namely the end of the image. Furthermore, the level of impulsivity and self-control could also affect the PoT judgment, as impulsive people are more likely to experience a slowing down of time during situations in which they are not able to act quickly and to fulfill their urge^19^. Therefore, the influence on the PoT of impulsivity personality traits but also that of anxiety, which is known to consume attentional resources^20^, were also examined in our study using the Barratt Impulsivity Scale and the State Anxiety Inventory (see method).

## Methods

### Participants

The final sample was composed of 803 undergraduate psychology students (M_age_ = 19.91, SD = 4.07, 551 Women) at the University of Clermont Auvergne (France) who participated in this experiment to validate course credits and after giving their informed consent. The experiment complied with the Declaration of Helsinki and was approved by the Research Ethics Committee of the University Clermont Auvergne (IRB00011540-2021-11).

### Material and procedure

Participants performed a single trial in which they saw an image of a black-and-white pattern. They triggered the presentation of the image by pressing a key on the computer keyboard after seeing the word “ready”. Before pressing, they read instructions explaining that they were going to see an image and that they would have to indicate their feelings when looking at this image. They then read that they would have to estimate the image duration in the prospective temporal judgment condition (N = 373), but not in the retrospective temporal judgment condition (N = 430). In the prospective condition, they were also instructed not to count because it would bias the scientific data^21^. After the temporal tasks, participants were asked a specific question about how they allocated their attention to time, thus making it possible to test the effectiveness of the retrospective and prospective instructions (I paid a lot of attention to time, from 1 disagree to 7 strongly agree). In each instruction condition (prospective vs. retrospective), participants were arbitrarily assigned to one of the experimental groups, which differed in terms of the range of durations to be judged, either in the range of seconds or minutes, as well as of the image duration. There were 3 image durations for each duration range, i.e., 20, 30 and 45 s for the short (s) durations and 80, 120 and 180 s for the long (min) durations. The latter durations were chosen as a multiple (× 4) of the former. These durations were defined on the basis of stimulus durations used in previous studies showing that there was no relationship between PoT judgment and duration judgment for short durations of up to about 40 s but a significant correlation for longer durations from 1 to 2 min. In addition, not very long durations were used in order to reduce the length of the experiment so that it would not be too boring^8,9,13,16^.

After the presentation of the image, all participants were asked to give their verbal estimation of its duration in seconds and were recalled that one minute is equal to 60 s (Duration Judgment).

They were then asked to indicate their experience of the passage of time (PoT judgment) by indicating their agreement with a series of 4 different statements on a 7-point Likert response scale ranging from disagree to strongly agree: (PoT_1_) I had the impression that time passed slowly (reverse score used); (PoT_2_) I had the impression that time passed quickly; (PoT_3_) I didn't notice time passing; (PoT_4_) What I saw passed quickly. Because we used Structural Equation Modeling (SEM) based on regressions with a latent variable that results from the variance and covariance of different observed variables (multiple manifest variables), we used 4 PoT items instead of a single item as previously used in the PoT studies. In addition, it allowed us to check for consistency of participants’ temporal judgment on several items. The internal consistency of participants’ responses to these 4 items was high, as indicated by Cronbach's alpha (α = 0.88).

After the temporal tasks, a series of questions were asked to assess participants’ experience in terms of image-directed attention, boredom and projection into the future (the upcoming end of the image) using the same 7-point Likert-type response scale. For attention, the statements were: (A_1_) What I saw had my full attention; (A_2_) My attention was focused on the image; (A_3_) I was focused on what I was seeing. For future projection, they were: (F_1_) I was mostly thinking about when the picture was going to end; (F_2_) My attention was focused on what was going to happen; (F_3_) I was constantly waiting for the next questions to come up; (F_4_) I was thinking about the future. And for boredom, they were: (B_1_) Looking at the picture, I was bored; (B_2_) What I saw was not interesting; (B_2_) I had trouble stopping myself being bored. Cronbach's alpha was good for each dimension tested (Attention, α = 0.91; Future, α = 0.80; Boredom; α = 0.85).

In addition, happiness and sadness were assessed using the subscales of the Brief Mood Introspective Scale (BMIS)^22^: for happiness: (H_1_) dynamic, (H_2_) happy, (H_3_) content; and for sadness: (T_1_) sad, (T_2_) melancholic, (T_3_) tired, (T_4_) exhausted. The scores on the arousal-calm mood subscale of the BMIS were also calculated. The consistency of participants’ responses on the items of each subscale was good (Happiness, α = 0.82; Sadness, α = 0.77; Arousal, α = 0.89).

In addition, to enable us to assess their level of self-reported anxiety, participants completed the 6-item short form of the State Anxiety Inventory (α = 0.74) (S-STAI)^23,24^, and to permit assessment of the impulsivity trait, they responded to the 15-item short form of the Barratt Impulsivity Scale (α = 0.77) (BIS)^25^. Impulsivity was assessed via 3 order factors: attentional, motor, and non-planning impulsivity. Attentional impulsivity is described as an inability to pay attention or focus, motor impulsivity as acting without thinking, and non-planning impulsivity as a lack of foresight or prior thought^25–27^.

### Statistical analyses

We used SPSS for our initial statistical analyses. A series of *t*-student tests were performed to compare the mean scores on demographic characteristics between participants in the prospective and the retrospective instruction condition. No significant differences were found between the groups (Table 1). Only mood was rated as more pleasant in the prospective than in the retrospective group (*p* < 0.05, Cohen’s *d* = 0.165). Regression-based analyses were then conducted using a General Linear Model (GLM) with the different time judgments as the dependent variable. For our models, two dependent variables were tested: time error [(time estimates-*t*)/*t*)] and the PoT judgment (average of responses on PoT_1,2,3,4_ items, with PoT_1_ reversed). In a first model, we examined the effect of stimulus durations (*StimD*) as the independent variable on our two variables, in each instruction condition taken separately (prospective and retrospective), to verify the discrimination of time in each condition (Y = *α* + β1*StimD + *ε)*. In all subsequent analyses, we did not test stimulus duration and tested duration ranges to simplify the analyses and in line with the SEM. In a second model, we tested the effects of the prospective vs. retrospective condition (*ProRetro*) and duration ranges (seconds and minutes) (*DurR*). As presented below, we included in this model individual scores on the specific question of attention devoted to time (*AtT*), which represents the factor underlying the difference between the prospective and the retrospective condition^14,15^. However, when this attention factor was included in the model, the instruction condition (prospective vs. retrospective) lost its predictive power. This suggests that even with the retrospective temporal judgment instruction condition, some participants paid attention to time, although to a lesser extent. Therefore, this attention-to-time factor was considered in our models rather than the prospective vs. retrospective condition, with the duration range factor, as well as their interaction (Y = *α* + β1*DurR + β2*AtT + β3*DurR*AtT + *ε*).

**Table 1:** Demographic characteristics in the prospective and the retrospective group for the age, the scores on the Short State Anxiety Inventory (S-STAI), the 3 factors of the Barratt Impulsivity Scale (BIS) and the different mood scores of the Brief Mood Introspective Scale (BMIS).

	Prospective		Retrospective		*t*	*p*
	M	SD	M	SD		
Age	20.19	3.931	19.66	4.18	1.76	0.08
S-STAI—Anxiety	12.85	5.602	12.91	5.8	0.16	0.87
BIS—Motor impulsivity	21.34	9.381	21.57	10.07	0.33	0.74
BIS—Non-planning impulsivity	21.9	6.452	21.94	7.25	0.08	0.94
BIS—Attention impulsivity	18.75	7.144	19.68	8.21	1.66	0.1
BMIS—Pleasant-Unpleasant mood	45.58	12.836	43.39	13.52	2.3	0.02
BMIS—Arousal-Calm mood	24.03	7.441	24.53	8.17	0.88	0.38
BMIS—Positive-Tired mood	14.08	5.008	13.79	5.13	0.79	0.43
BMIS—Negative-Relaxed mood	9.72	3.656	10.09	3.91	1.38	0.17

We then used AMOS for Structural Equation Modelings (SEM) to analyze the relationships between the variables influencing each type of temporal judgment (PoT or duration judgment, i.e., time error) for each duration range taken separately. As described in the results, initial correlation analyses were performed and resulted in the exclusion of non-significant variables (arousal, anxiety, attention-impulsivity, motor-impulsivity). The final model included 23 observed variables (PoT_1,2,3,4_; H_1,2,3_; T_1,2,3,4_; B_1,2,3_; A_1,2,3_; F_1,2,3,4_; Non-planning Impulsivity score; Time Error) and 6 latent variables (PoT, Boredom, Happiness, Sadness, Attention, Future). The observed variables used were those defined as representing the latent variable without prior empirical adjustment. Values of CFI > 0.90 and RMSEA < 0.08 indicated an acceptable model fit, and RMSEA < 0.05 indicated a good fit^28–30^.

## Results

**Figure 1:**
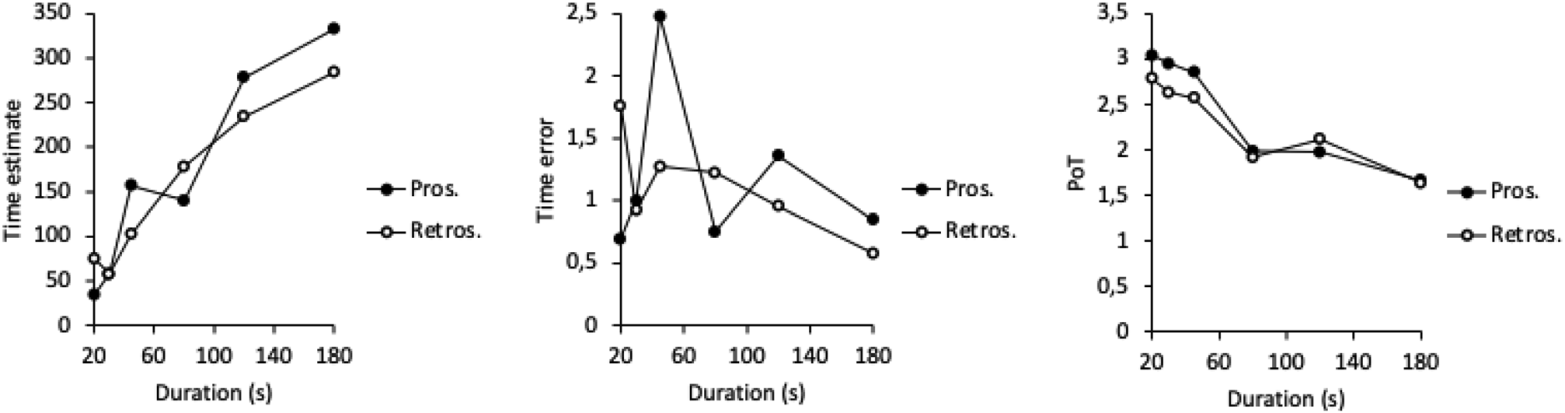
Temporal judgments as a function of stimulus durations (s) in the prospective and the retrospective conditions for the seconds and the minutes: Time estimate in seconds; Time error (time estimates-*t*)/*t*); and Passage-of-Time judgment.

The first GLM conducted to verify the effect of the stimulus duration on the temporal judgments confirmed a significant stimulus duration effect on the PoT judgment in the prospective condition (E = − 0.31, ES = 0.04, 95%CI [− 0.39; − 0.23], *β* = − 0.37, *t* = − 7.59, *p* < 0.0001), as well as in the retrospective condition (E = − 0.22, ES = 0.03, 95%CI [− 0.28; − 0.16], *β* = − 0.32, *t* = − 6.93, *p* < 0.0001) (Table 2). Therefore, the longer the stimulus duration, the more the participants experienced time as slowing down (Fig. 1). As illustrated Fig. 1, there was also an effect of stimulus duration on time estimates in both the prospective and the retrospective condition (E = 61.34, ES = 9.10, 95%CI [43.44; 79.24], *β* = 0.33, *t* = 6.74, *p* < 0.0001; E = 43.54, ES = 7.45, 95%CI [28.89; 58.18], *β* = 0.27, *t* = 5.84, *p* < 0.0001, respectively). However, the time error (TE) indicated that the participants generally tended to overestimate the stimulus durations, *t*(808 = 6.80, *p* < 0.0001. In addition, the TEs were quite similar for the different durations in the prospective condition (E = − 0.005, ES = 0.14, 95%CI [− 0.29; 0.28], *β* = − 0.002, *t* = − 0.04, *p* = 0.97), while they tended to increase as the duration length decreased in the retrospective condition (E = − 0.37, ES = 0.19, 95%CI [− 0.73; − 0.002], *β* = − 0.10, *t* = − 1.98, *p* = 0.049).

**Table 2:** Mean (SD) of PoT judgment for the different stimulus durations in the prospective and the retrospective condition.

Stimulus duration	Prospective		Retrospective	
	M	SD	M	SD
20	3.04	1.53	2.78	1.36
30	2.95	1.56	2.63	1.27
45	2.85	1.59	2.57	1.14
80	1.98	0.86	1.91	0.71
120	1.97	0.99	2.11	1.03
180	1.66	0.66	1.63	0.61

**Figure 2:**
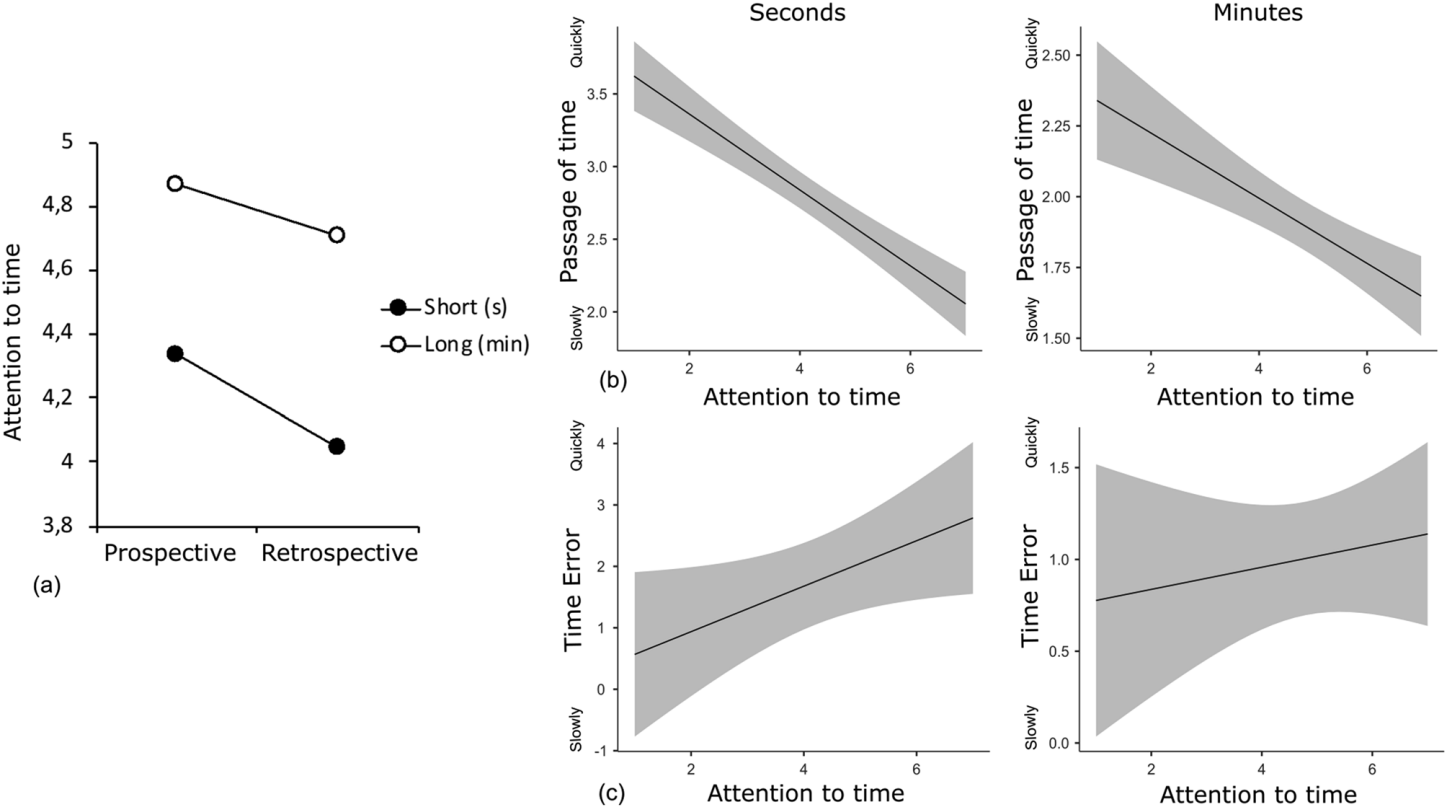
Attention to time in the prospective and the retrospective condition, and relationship between attention to time and the Passage-of-Time judgment and time error for the short durations of seconds and the long durations of minutes.

A GLM was then performed on the TE and the PoT judgment with the instruction condition (prospective vs retrospective), the short (s) vs long (min) duration range, and attention to time entered as factors. The results revealed that the level of attention to time was a better predictor of variation in temporal judgments (both TE and PoT) than the instruction condition per se. Indeed, when entered in the model with attention to time and duration range, the prospective vs. retrospective condition lost its significant predictive power (all *p* > 0.05). Indeed, the level of attention devoted to time tended to be lower in the retrospective than in the prospective condition (Fig. 2a, E = − 0.26, ES = 0.137, 95%CI [− 0.531; 0.009], *β* = − 0.07, *t* = − 1.0, *p* = 0.058). This result suggests that even in the retrospective condition some participants paid attention to time, although to a lesser extent. Therefore, we excluded the prospective vs retrospective factor from the model and carried out the GLM with the attention-to-time factor and the duration range as variables. The GLM on the PoT judgment showed a significant main effect of the attention-to-time factor (E = − 0.197, ES = 0.022, 95%CI [− 0.24; − 0.15], *β* = − 0.29, *t* = − 8.93, *p* < 0.0001) and the duration range (E = − 0.780, ES = 0.083, 95%CI [− 0.94; − 0.62], *β* = − 0.31, *t* = − 9.35, *p* < 0.0001), as well as a significant interaction between these two factors (E = 0.15, ES = 0.045, 95%CI [0.06; 0.24], *β* = 0.11, *t* = 3.24, *p* < 0.0001). Attention to time was indeed found to be a significant predictor of PoT for both the short (E = − 0.26, ES = 0.03, 95%CI [− 0.33; − 0.20], *β* = − 0.36, *t* = − 7.98, *p* < 0.0001) and the long durations (E = − 0.12, ES = 0.03, 95%CI [− 0.17; − 0.07], *β* = − 0.24, *t* = − 4.51, *p* < 0.0001) (Fig. 2b). The GLM on the TE also showed a main effect of attention-to-time factor (E = 0.234, ES = 0.12, 95%CI [0.008; 0.46], *β* = 0.08, *t* = 2.04, *p* = 0.042) and of duration (E = − 0.86, ES = 0.43, 95%CI [− 1.71; − 0.01], *β* = − 0.7, *t* = − 1.98, *p* = 0.048) but no attention-to-time x duration interaction (E = − 0.31, ES = 0.24, 95%CI [− 0.80; 0.15], *β* = − 0.05, *t* = − 1.32, *p* = 0.19) (Fig. 2c). Therefore, the more participants paid attention to time, the more time was overestimated, regardless of duration range. In sum, when the participants paid little attention to time, as was more the case in the retrospective condition, time seemed to pass faster, especially for short durations (Fig. 2b). Time was also found to be perceived as shorter when attention to time decreased (Fig. 2c).

To identify the best predictors of both PoT judgment and duration judgment, but also those common to these two types of temporal judgment, the same structural equation model was used with the same number of observations and latent variables. Individual anxiety scores on the S-STAI and those on the BMIS calm-arousal scale were not included in the model as primary analyses did not show relevant correlations between temporal judgments and either arousal or anxiety level (Arousal: PoT: *r* = 0.004, *p* = 0.91; TE: *r* = − 0.06, *p* = 0.08; Anxiety: PoT: *r* = 0.02, *p* = 0.60; TE: *r* = − 0.07, *p* = 0.44). In addition, only the non-planning impulsivity scores were included in the model because the correlations between the temporal judgments and the other impulsivity dimensions were not significant or weaker (non-planning impulsivity; PoT: *r* = 0.03, *p* = 0.49; TE: *r* = − 0.16, *p* < 0.001; motor impulsivity: PoT: *r* = 0.005, *p* = 0.88; TE: *r* = − 0.088, *p* = 0.01; attentional impulsivity: PoT: *r* = − 0.02 *p* = 0.58, TE: *r* = − 0.10, *p* = 0.01).

**Figure 3:**
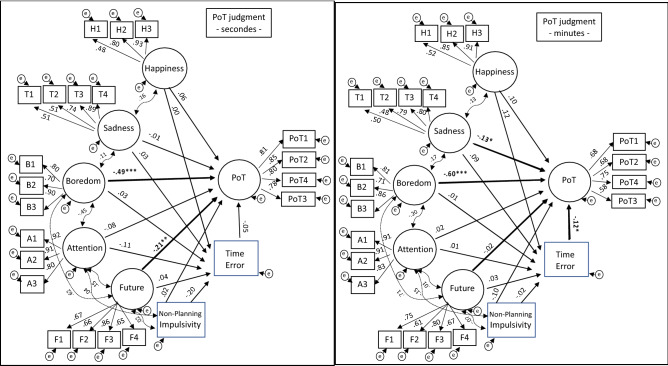
Structural Equation Model analyzing the relationships between variables thought to influence the Passage-of-Time judgment for the short (seconds) (left panel) and long (minutes) duration (right panel).

**Figure 4:**
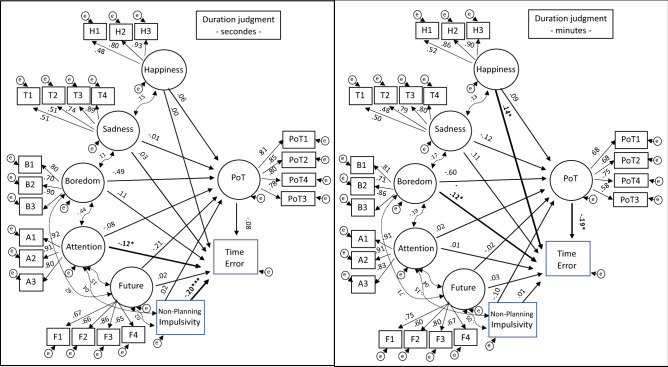
Structural Equation Model analyzing the relationships between variables thought to influence the time error in the verbal duration judgment task for the short (seconds) (left panel) and long (minutes) duration (right panel).

Figure 3 and Fig. 4 show the structural models for PoT judgment and time error, respectively. SEM indicates that the fit of the model to the data is good or acceptable for PoT judgment in each duration condition (Short duration (s): X^2^ (212) = 510.78, CFI = 0.936, RMSEA = 0.056; Long duration (min): X^2^ (212) = 502.43, CFI = 0.912, RMSEA = 0.062) and TE (Short: X^2^ (212) = 509.17, CFI = 0.936, RMSEA = 0.056; Long: X^2^ (212) = 499.39, CFI = 0.913, RMSEA = 0.062).

The structural models suggest that the best predictor of PoT judgment was the level of boredom experienced, regardless of whether the durations lasted a few seconds (ß = − 0.49, *p* < 0.0001) or several minutes (ß = − 0.60, *p* < 0.0001) (Fig. 3). Therefore, the feeling of passage of time was mainly explained by the subjective feeling of boredom while viewing the image. Although to a lesser extent, two other factors were also significant predictors of the PoT judgment: projection into the future (the end of the image) for short durations (ß = − 0.21, *p* = 0.002) and sadness for long durations (ß = − 0.13, *p* = 0.045). The emotions experienced were therefore the main predictors of PoT judgment, especially for durations of several minutes. Obviously, as discussed later, boredom covaried with both the emotion of sadness and attention directed to the image, although the magnitude of the covariance with emotion increased, and that with attention decreased for the long durations of several minutes compared to the short durations of a few seconds (sadness: ß_short_ = 0.11, *p* < 0.05, ß_long_ = 0.17, *p* < 0.05; attention ß_short_ = − 0.45, *p* < 0.05, ß_long_ = − 0.20, *p* = 0.05).

In contrast, the predictors of duration judgment (TE) differed significantly between short and long durations. For durations of a few seconds, the main predictors of time distortion were attention-related factors: the amount of attention allocated to the image (ß = − 0.12, *p* = 0.04), and participants’ attentional traits in terms of impulsivity (ß = − 0.20, *p* < 0.0001) (Fig. 4). For long durations of several minutes, these factors ceased to be significant and were replaced by the effect of the emotions experienced, namely boredom (ß = − 0.12, *p* = 0.03), and the decrease in happiness (ß = 0.14, *p* = 0.02), in the same way as for the PoT judgment. Indeed, duration judgment and PoT judgment became related for long durations of several minutes (ß = − 0.19, *p* < 0.0001), whereas they were not related for short durations (ß = − 0.079, *p* = 0.227).

## Discussion

The results indicated that the participants tended to overestimate the stimulus duration in our experimental study. They did, however, replicate the results of studies using short durations (seconds) by showing that, in a verbal duration estimation task, the stimulus duration was judged longer in the prospective condition compared to the retrospective condition, specifically, when the self-reported level of attention allocated to time increased^31–33^. Indeed, in our study, in contrast to previous studies, we controlled for the level of attention devoted to time and found that it was the main factor that explained time judgments rather than the instruction condition itself (retrospective vs. prospective), with less attention being paid to time in the retrospective than in the prospective condition. The SEM corroborated this finding by showing that the best predictors of the short duration judgment were the amount of attention allocated to the image but also individual attention-related abilities: the more impulsive the participants were, the shorter the stimulus duration was estimated to be.

The differences in PoT judgment between these 2 experimental conditions (prospective vs. retrospective) have never been tested in the same study. Our results thus showed for the first time that the temporal pattern for the PoT judgment was similar to that found for the duration judgment, with time being reported as passing more quickly in the retrospective than in the prospective condition, more precisely when less attention resources were allocated to time processing. However, the SEM suggested that the factor explaining the inter-individual differences in the PoT judgment was not related to attention as for the duration judgments, but to the emotion experienced while viewing the image on the computer screen, namely the boredom felt. This was consistent with the results of studies showing that emotion, especially boredom (but also sadness/happiness), plays a key role in everyday-life passage-of-time judgments (e.g.^12,17,34–37^). However, in the present experimental condition, participants’ projection into the future (i.e., the end of the image) also emerged as a significant predictor of PoT, although to a lesser extent (see also^18^). Furthermore, the level of boredom covaried not only with emotions of negative valence (sadness) but also with the level of attention directed towards the image. This highlights the two intertwined facets of boredom, i.e., the affective and cognitive facets. Eastwood et al.^38^ defined boredom as “an aversive state when we are not able to successfully engage attention with internal (e.g., thoughts or feeling) or external (e.g., environmental stimuli) information” (p. 482). However, our results showed that the self-reported level of attention did not directly predict the PoT judgment. This suggests that the PoT judgment is primarily the result of participants’ introspective analysis of their emotional state (the affective experience of boredom), which manifestly depends on the cognitive activity required to perform the task. The PoT is thus related to the minimal self, i.e., self-consciousness during an activity expressed in temporal terms as an extension or contraction of self-duration^7,16,39^.

Our results showed that when the duration increased and exceeded one minute, the role of projection into the future and of the boredom-attention link in the PoT judgment decreased. Emotion, i.e., the boredom felt and its affective dimension (sadness), then prevailed. Similarly, the attention-related factors that explained the variance in the judgment of short durations lost their predictive power in favor of emotion for long durations. For durations of the order of minutes, the more boredom increased, the sadder participants felt, and the greater the time errors (as overestimates of elapsed time) were. Therefore, long durations of several minutes were judged longer, and the passage of time seemed to go more slowly.

Judgments of durations and of PoT were therefore linked for long durations of several minutes, whereas this was not the case for short duration of a few seconds. For values of the order of minutes, PoT judgment was thus a significant predictor of duration judgment and vice versa. This link that emerged between PoT and duration judgment is consistent with the results found by Droit-Volet et al.^13^ with durations going from 2 to 32 min. There is thus a growing body of data in support of the theory according to which memory mechanisms prevail in judgments of long durations, as the influence of the awareness of time and its passage increases. Consequently, since PoT depends on situational context and its emotional effects, mechanisms associated with the storage and the retrieval of non-temporal information in long-term memory are involved in temporal judgments, as suggested by the studies on retrospective time. However, as explained in the theory of explicit judgments of very long durations^13^, this does not mean that long durations are not, for example, partly encoded and stored in long-term memory as a sequence of shorter durations. The recent models of recurrent neuronal networks consider this type of dynamic mechanism to be the basis of temporal encoding^40,41^. Furthermore, distortions of duration might also be observed due to duration consolidation processes in long-term memory^42,43^. In line with this conjecture, our studies clearly found a significant effect of stimulus durations in temporal judgments, even with long durations and even though temporal discrimination was reduced for long durations. However, this decrease in temporal discrimination of long durations may also be due to the verbal symbolic measure used by humans (verbal response), namely minutes, which reduces the differences between durations through a process of verbal attraction to minutes (1 min, 2 min, 3 min, etc.)^44^.

Our study replicated the results of studies on PoT judgment and verbal duration judgment and clearly confirmed the differences and similarities between these two types of judgment depending on the length of the durations, i.e., greater than or less than one minute. Thus, the results in our study make a contribution and are consistent with the theory according to which PoT judgment results from participants’ introspective analyses of their affective states depending on the situational context encountered and the cognitive activity performed^7,16^. This highlights the importance of further investigating awareness of self-duration, expressed in terms of passage of time, in judgments of long durations and their relationships to judgments of short durations in order to develop a general model of judgments of time. However, before such a theory could be proposed, other ranges of durations need to be tested with our experimental procedure, such as longer durations of 15 or 30 min, so as not to be limited, as in our study, to durations of the order of tens of seconds and 1–3 min. Controlling for the degree of attention paid to time in the retrospective condition, we also found that some participants thought about time, although to a lesser in the retrospective than in the prospective condition. In the context of studies comparing prospective and retrospective time judgment, it would be important to directly test in the same experiment the role of the degree of attention given to time and its effects on both the duration and the PoT judgment for different duration values.

## Data Availability

Data supporting the findings of this study are available from the first author Natalia Martinelli (natalia.martinelli@uca.fr) upon reasonable request.
